# Can pulsatile CSF flow across the cerebral aqueduct cause ventriculomegaly? A prospective study of patients with communicating hydrocephalus

**DOI:** 10.1186/s12987-019-0159-0

**Published:** 2019-12-23

**Authors:** P. Holmlund, S. Qvarlander, J. Malm, A. Eklund

**Affiliations:** 1 Department of Radiation Sciences, Umeå University, Umeå, Sweden; 2 Department of Pharmacology and Clinical Neuroscience, Umeå University, Umeå, Sweden; 3 Umeå Centre for Functional Brain Imaging, Umeå University, Umeå, Sweden

**Keywords:** Communicating hydrocephalus, Computational fluid dynamics, Cerebrospinal fluid pressure, Brain imaging, Cerebral aqueduct

## Abstract

**Background:**

Communicating hydrocephalus is a disease where the cerebral ventricles are enlarged. It is characterized by the absence of detectable cerebrospinal fluid (CSF) outflow obstructions and often with increased CSF pulsatility measured in the cerebral aqueduct (CA). We hypothesize that the cardiac-related pulsatile flow over the CA, with fast systolic outflow and slow diastolic inflow, can generate net pressure effects that could source the ventriculomegaly in these patients. This would require a non-zero cardiac cycle averaged net pressure difference (Δ*P*_*net*_) over the CA, with higher average pressure in the lateral and third ventricles.

**Methods:**

We tested the hypothesis by calculating Δ*P*_*net*_ across the CA using computational fluid dynamics based on prospectively collected high-resolution structural (FIESTA-C, resolution 0.39 × 0.39 × 0.3 mm^3^) and velocimetric (2D-PCMRI, in-plane resolution 0.35 × 0.35 mm^2^) MRI-data from 30 patients investigated for communicating hydrocephalus.

**Results:**

The Δ*P*_*net*_ due to CSF pulsations was non-zero for the study group (p = 0.03) with a magnitude of 0.2 ± 0.4 Pa (0.001 ± 0.003 mmHg), with higher pressure in the third ventricle. The maximum pressure difference over the cardiac cycle Δ*P*_*max*_ was 20.3 ± 11.8 Pa and occurred during systole. A generalized linear model verified an association between Δ*P*_*net*_ and CA cross-sectional area (p = 0.01) and flow asymmetry, described by the ratio of maximum inflow/outflow (p = 0.04), but not for aqueductal stroke volume (p = 0.35).

**Conclusions:**

The results supported the hypothesis with respect to the direction of Δ*P*_*net*_, although the magnitude was low. Thus, although the pulsations may generate a pressure difference across the CA it is likely too small to explain the ventriculomegaly in communicating hydrocephalus.

## Background

This paper investigates the potential pressure difference over the communicating cerebral aqueduct, a feature that may have a role in cerebral ventricular enlargement (or ventriculomegaly) in communicating hydrocephalus. Ventriculomegaly in hydrocephalus is thought to be due to brain tissue deformation as the result of an active distension of the ventricles [1], indicating a pressure gradient between the ventricles and the subarachnoid space (i.e. a transmantle pressure gradient). To generate such a pressure gradient, a pressure difference over the cerebral aqueduct (CA) is expected, since the CA connects the ventricles and the subarachnoid space. If the CA is obstructed (non-communicating hydrocephalus) it is reasonable that the bulk flow generated by the CSF production in the choroid plexus could be sufficient to produce a pressure difference and ventriculomegaly. However, in communicating hydrocephalus no apparent flow obstruction is shown on radiology [1] leaving the question of a pressure difference open.

In addition to bulk flow, CSF flows in a to and fro movement across the CA, synchronized with the heartbeat [2]. A typical characteristic of communicating hydrocephalus is high pulsatile CSF flow [3–5], and experimental studies have hinted at a causal link between altered CSF pulsations and ventricular enlargement [6–8]. Since the largest pressure changes in the ventricular system occur over the CA [9, 10], it is reasonable that altered pulsatile flow dynamics over the CA could play a part in the development of ventriculomegaly in these patients. Looking at the flow through the CA, it can be observed that it is asymmetric, i.e. higher and more rapidly varying during systolic CSF outflow and lower and more gradually varying during diastolic inflow [11]. Our hypothesis is that this asymmetry in the pulsatile flow over the open CA can generate a net transmantle pressure gradient, one that, over time, could potentially contribute to the ventriculomegaly in communicating hydrocephalus.

In addition to viscous flow resistance as described by the Poiseuille equation, flow through constrictions such as the CA will result in additional pressure losses. These pressure losses occur in places where the geometry is changing rapidly, resulting in recirculation zones, i.e. vortex formations, where fluid energy is dissipated [12, 13]. In the CA this can occur at the entrance and exit to the third and fourth ventricles due to enlargement/contraction of the flow geometry. These contraction and enlargement (or discharge) effects are not only dependent on *geometrical shape* but are also *non*-*linearly dependent on the flow rate* going into the CA [12, 13]. This means that even with zero net flow the asymmetrical pulsations through the CA can generate net pressure differences/gradients across the CA over each cardiac cycle. This is in contrast to the resistive losses described by Poiseuille, which amount to a net pressure difference of zero if the pulsatile flow has a zero net flow. Over time, such a net transmantle pressure gradient could potentially contribute to ventriculomegaly. This net pressure gradient and thus the net pressure difference (Δ*P*_*net*_) across the CA must be positive (i.e. with higher pressure in the third ventricle) to explain the enlargement of the third and lateral ventricles.

It should be mentioned that in vivo studies of communicating hydrocephalus patients have not been able to measure a transmantle pressure gradient (or pressure difference) [14, 15], indicating that the corresponding Δ*P*_*net*_ is likely small in magnitude. In a biomechanical modeling study Dutta-Roy et al. predicted that a transmantle pressure difference of 235.44 Pa (1.764 mmHg) was needed to produce the ventricular expansion and brain alterations seen in normal pressure hydrocephalus, acting over the course of a few days (at most) [16]. Since ventriculomegaly may develop over a much longer time in communicating hydrocephalus (over several years) the required transmantle pressure difference can be expected to be even lower. A possible way of detecting and quantifying this pressure difference is by computational fluid dynamics (CFD), which provides a useful tool for identifying pressure behavior that is impossible to assess using in vivo pressure sensors. CFD also allows us to take the entire CA geometry into account, which is problematic to do analytically. Previous CFD studies have presented data on the pressure differences over the CA in hydrocephalus case studies and in healthy [9, 10, 17, 18], but to fully determine its magnitude and potential role in communicating hydrocephalus a study specifically designed to investigate the transmantle pressure gradient in a larger group of communicating hydrocephalus patients is needed, utilizing the highest quality imaging data feasible.

In order to evaluate our hypothesis, the aim of this study was to calculate Δ*P*_*net*_ across the CA utilizing CFD based on prospectively collected high-resolution structural and velocimetric magnetic resonance imaging (MRI) data from 30 patients investigated for communicating hydrocephalus.

## Methods

### Bench test of feasibility

**Fig. 1:**
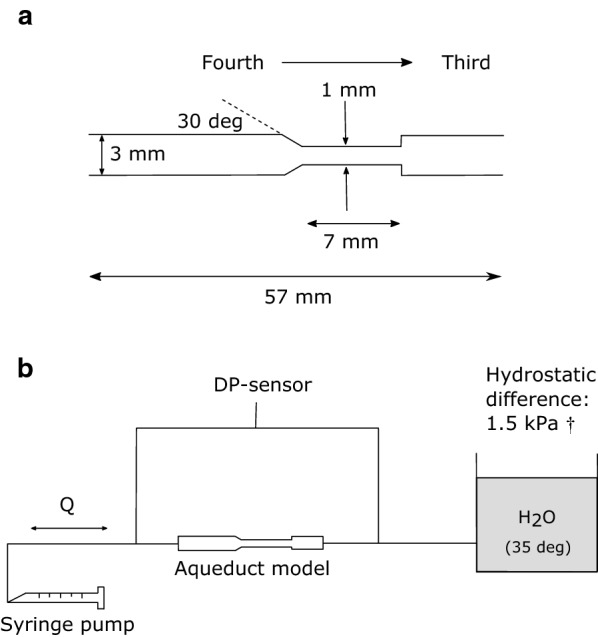
Illustration of the CA bench-model (**a**) and the experimental setup (**b**). The pump was controlled using *LabVIEW* (National Instruments Corporation, Austin, Texas, US), to recreate aqueductal pulsatile flows based on CA flows measured in 43 healthy elderly and 22 idiopathic normal pressure hydrocephalus (INPH) patients [11]. Q is the pulsatile flow rate, DP is Differential pressure. ^†^Reference value for intracranial pressure in healthy in the supine position [31]

Prior to the prospective patient study, bench-tests were performed to test the feasibility of the hypothesis. The bench setup consisted of a simplified aqueduct model, a differential pressure (DP) transducer (Druck LPM 8381, General Electric, US), a programmable syringe pump, and a container of water representing the third ventricle (Fig. 1). The pressure difference across the model was measured while the model was subjected to pulsatile aqueductal flows produced by the syringe pump. These flows were based on phase contrast MRI data from INPH patients and healthy elderly (from [11]). The net flow was removed to only investigate the effects of the pulsations. The resulting flow rate was documented by measuring the syringe pump position using a position sensor (F58000136 linear position Transducer, LT series, Honeywell, US). The system was kept horizontal and the temperature of the water was kept close to body temperature (acquired temp: 35°C). Net pressure over the model, Δ*P*_*net bench*_, was calculated by averaging the recorded differential pressure over 30 cardiac cycles. Each measurement was repeated six times. The experiments were recreated by applying the acquired experimental flow rates and the model geometry to CFD simulations to estimate Δ*P*_*net bench CFD*_.

### Study subjects

The prospective patient study included thirty-two patients referred to the neurological department at Umeå University for investigation of communicating hydrocephalus during the time period from October 2016 to May 2017. All patients had enlarged cerebral ventricles, defined as an Evan’s ratio $\ge$ 0.3, and symptoms of normal pressure hydrocephalus (NPH) (i.e. gait disturbances, cognitive decline and/or urinary incontinence). The preoperative investigation included case history, neurological examination, blood chemistry, CSF dynamic investigations and MRI (including sequences specified below). Patients with obstruction of the CA based on the anatomical MRI sequences were excluded (we also verified that the median aperture, i.e. foramen of Magendie, was open). Two patients were excluded from the analysis: one with CA obstruction, and one with severely increased intracranial pressure (3.8 kPa or 28.5 mmHg), resulting in a total of n = 30 patients. Out of the thirty patients, 15 were diagnosed as INPH [19], 9 got a different hydrocephalus diagnosis (e.g. secondary causes), and 6 were diagnosed with diseases other than hydrocephalus (mainly dementias, but still with enlarged ventricles and a communicating CA). Group characteristics are presented in Table 1.

**Table 1:** Characteristics of the communicating hydrocephalus group

Parameter	Mean $\pm$ SD	Range
Subjects	30 (total)	–
INPH	15	–
Communicating hydrocephalus	9	–
Other	6	–
Sex (female/male)	14/16	–
Age (years)	75 $\pm$ 6	60–86
MMSE	24 $\pm$ 5	15–30
Evans’ index	0.37 $\pm$ 0.04	0.31–0.47
Heart rate (bpm)	72 $\pm$ 13	47–98

### Magnetic resonance imaging

MRI investigations were carried out on a 3 Tesla scanner (GE Discovery MR 750, Waukesha, WI) with a 32-channel head coil. The structural data for the ventricular system was acquired by a Fast Imaging Employing Steady-state Acquisition Cycled Phases (FIESTA-C) sequence, a sequence with T2/T1 contrast which allows for excellent distinction between fluid-filled spaces and surrounding tissues [20, 21]. The sequence was optimized to achieve the highest structural resolution possible to avoid partial volume effects and to assess the CA geometry as accurately as possible. The settings were: repetition time/echo time 6.5/2.6 ms, flip angle was 55°, acquisition duration roughly 7–8 min, 3 signal averages, imaging volume 20 × 20 × 3.6 cm^3^, acquisition matrix 512 × 512 × 60. The FOV included the CA, the third and the fourth ventricles, but not the entirety of the lateral ventricles. The acquisition resolution was thus 0.39 mm × 0.39 mm in-plane, with slice thickness 0.6 mm. The slices were interpolated, resulting in a final resolution of 0.39 mm × 0.39 mm × 0.30 mm.

**Fig. 2:**
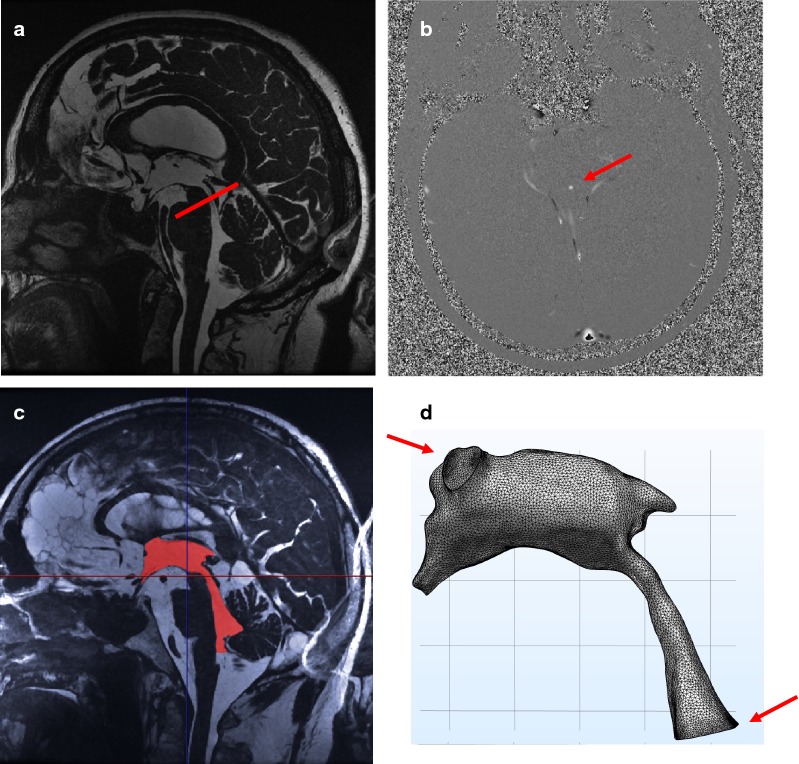
Illustration of the MRI flow measurements and mesh generation. **a** The FIESTA acquired data with indications for the PCMRI plane position (red line). **b** The resulting PCMRI (velocity) image in the plane across the aqueduct. **c** Segmentation of the ventricular system (red). **d** The constructed mesh with the defined flow boundaries (red arrows)

The velocity data was acquired by 2D-PCMRI with a plane positioned through the CA (Fig. 2a, b). The settings were: repetition time/echo time 9.8/5.6 ms, flip angle 6°, acquisition duration roughly 5–6 min, 3 signal averages, imaging volume 18 × 18 cm^2^, acquisition matrix 512 × 512, slice thickness 4.0 mm, arrhythmia rejection window 20%, 4 views per segment, and velocity encoding was 20 cm/s. The spatial in-plane acquisition resolution was thus 0.35 mm × 0.35 mm. Since partial volume effects are known to overestimate CA flow rates [22] we aimed for high resolution images for this sequence as well. The use of 3 signal averages was used to remedy the potential decrease in the signal to noise ratio that comes with the increased resolution. A peripheral pulse detector allowed for retrospective electrocardiography gating during data reconstruction, and data was reconstructed for 32 images over the cardiac cycle.

### Reconstruction of patient-specific geometries

Patient-specific geometries were segmented from the FIESTA sequence data (Fig. 2c) using Synopsys’ Simpleware™ software (Version M-2017.06; Synopsys Inc., Mountain View, USA). PH performed manual segmentations with guidance from an experienced neuroradiologist. Smoothing of the segmented volumes was then performed and a built-in mask statistics function was used to compare the volume of the aqueduct before and after smoothing to minimize potential changes in volume introduced by the smoothing operation. The volume of the aqueduct was not allowed to change more than 2% for any subject (group mean: + 1.0 ± 0.8%), while the entire ventricular system did not change more than 4% (group mean: + 1.5 ± 1.0%). The foramina of Monro were set as cranial outlet boundaries, while a plane in the fourth ventricle located just superior to the lateral and median apertures was set as the inlet boundary (Fig. 2d). The volumes were then exported to the CFD software.

### MRI flow velocity analysis

Analyses of the PCMRI measurements were performed using Segment (version 2.1 R5960, http://medviso.com/segment/) [23]. A region of interest (ROI) was drawn around the cross-section of the aqueduct in the magnitude images, with constant size and position for all 32 time frames. Segment’s built-in eddy current correction was utilized for all measurements. Averaging the velocity magnitudes of all pixels within the ROI and multiplying with the cross-sectional area yielded the measured flow rate for each time frame. The same operator performed all PCMRI analyses. However, fifteen subjects were independently analyzed by a second operator to assess the inter-rater variability of the PCMRI analyses.

### Computational fluid dynamics

CFD simulations were performed with COMSOL Multiphysics utilizing the finite element method (COMSOL Multiphysics^®^, version 5.3a, www.comsol.com, COMSOL AB, Stockholm, Sweden). Since CSF is an incompressible Newtonian fluid [24], the CSF fluid and pressure dynamics are described by the non-linear Navier–Stokes equations for incompressible flow:

$$\rho \frac{{\partial \varvec{u}}}{\partial t} + \rho \left( {\varvec{u} \cdot \nabla } \right)\varvec{u} - \nabla \cdot \left[ { - P\varvec{I} + \mu \left( {\nabla {\mathbf{u}} + \left( {\nabla {\mathbf{u}}} \right)^{\text{T}} } \right)} \right] = 0$$

$$\nabla \cdot \varvec{u} = 0$$

where ***u*** and *P* represent the CSF velocity and pressure that we want to solve for. The density was set to $\rho =$ 1000 kg/m^3^ and the viscosity to $\mu = 0.9 \times 10^{ - 3} \;{\text{Pa}}\;{\text{s}}$ [24]. Since flow is expected to be laminar within the ventricular system [17], the built-in laminar flow physics model was used. To solve the Navier–Stokes equations we used a non-linear solver based on Newton’s method, utilizing a fully coupled approach. The time stepping was handled using COMSOL’s backward differentiation formula (BDF) time-solver, an implicit solver based on the backward Euler method, with second order accuracy.

The segmented structural volumes were imported to COMSOL with the geometry simplification factor kept to a minimum (0.001). The meshes were constructed in COMSOL by using the physics-controlled setting. Boundary conditions were: no-slip on the ventricular and aqueduct walls, open boundary at the two intraventricular foramina (i.e. stress normal to the boundary was set to zero), and laminar parabolic inflow in the fourth ventricle (Fig. 2d). All surfaces were modeled as rigid. The flow rate measured in the CA was applied at the inflow boundary.^1^ The 32 points for each flow curve were interpolated using cubic splines. The inflow was ramped up from 0 to 1 during the simulations’ first 0.1 s. The time step was set to $\Delta {\text{t}} = 10^{ - 3} \;{\text{s}}$ and each simulation ran for 3 cardiac cycles. Since the results were highly similar for the second and third cycles, the third cycle was used for analysis. The simulations ran on an iMac (2.93 GHz Intel Core i7) and the simulation took roughly 48 h per subject.

Since we specifically aimed to investigate the effects of the flow pulsations, the net flow was removed for all simulations. Omitting the net flow was further motivated by the fact that repeated measurements at different points in time have shown high variability in the aqueductal net flow (intraclass correlation coefficient (ICC) 0.41 [22]). In contrast, repeated measurements for aqueductal stroke volume (SV) (i.e. pulsatile flow) is high (0.96 for SV [22]).

**Fig. 3:**
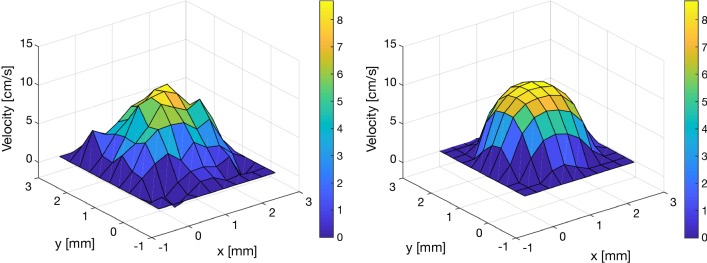
Comparison of PCMRI (left) and CFD velocity profiles (right) at maximum CA outflow in an example subject. The maximum spatial velocity for the group was 11.1 $\pm$ 5.3 cm/s for the PCMRI measurements and 11.5 $\pm$ 6.9 cm/s for the CFD simulations (mean difference: − 0.4 $\pm$ 4.4 cm/s, PCMRI-CFD). Generally, the largest differences were found for small aqueducts or aqueducts with the highest SV. The small difference could be partly attributed to the difference in spatial resolution between the PCMRI data and the CFD meshes (CFD had higher resolution). In this figure, the CFD data has been down-sampled spatially in order to simplify the visual comparison

To verify that our choice of inlet did not significantly alter the velocities in the CA, the maximum velocities during the time of maximum outflow from the ventricles were compared between the raw PCMRI-data and the corresponding plane in the CFD simulations. The velocity distribution for a typical case is presented in Fig. 3.

### Sensitivity analysis for the CFD

We performed a mesh sensitivity analysis by testing three meshes of different coarseness for 5 randomly selected subjects/cases, evaluating the change in the maximum pressure difference Δ*P*_*max*_ (Table 2). Since the relative error between the two finer meshes was only 2 ± 2% (with only one out of five cases exceeding 2%), the mesh of intermediate coarseness was used throughout as it severely reduced the computational time. For the main analysis (n = 30), the average number of mesh elements was 5.5 × 10^5^ ± 3.1 × 10^5^. The average and minimum mesh element quality (based on equiangular skew) was 0.67 ± 0.004 and 0.10 ± 0.04, respectively.

**Table 2:** Mesh sensitivity analysis for the maximum pressure difference Δ*P*_*max*_ across the CA (presented in Pa)

Test subject	Coarsest mesh	Intermediate mesh	Finest mesh
1	66.0	65.9	66.4
2	18.2	18.6	18.3
3	25.2	20.9	19.6
4	23.2	19.8	19.7
5	38.9	34.5	35.2

Coarsest, intermediate and finest corresponded to mesh elements roughly of the order 10^5^, 4 × 10^5^ and 10^6^, respectively. The intermediate mesh was the one chosen for the main analysis

### Patient specific pressure difference

The Δ*P*_*net*_ was calculated as the time-average of the pressure difference between the fourth ventricle and the foramen of Monro over the cardiac cycle. The local pressures in these two locations were calculated as the average across the inlet boundary (in the fourth ventricle) and outlet boundaries (the foramina of Monro), respectively. Since the variations in intracranial pressure over the cardiac cycle are a few hundred Pa [25], we expect the Δ*P*_*net*_ to have to be at least of the order of 1 Pa in order to be meaningful.

**Fig. 4:**
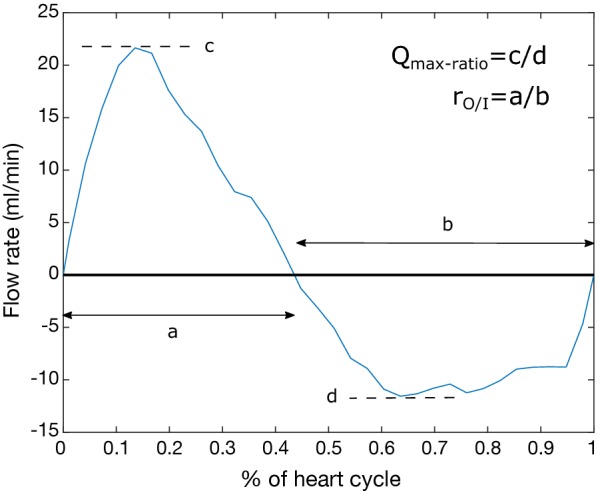
A CA flow curve visualizing the asymmetry ratios for CA out- and inflow

We also investigated the dependency of Δ*P*_*net*_ on different flow and geometrical properties, in order to test how different characteristics contribute to the Δ*P*_*net*_. The characteristics investigated were: mid CA cross-sectional area, aqueductal SV, and CA flow asymmetry. The CA flow asymmetry was described in two different ways: the maximum outflow/inflow ratio (*Q*_*O/I*_) and the ratio of time duration of outflow vs. inflow (*r*_*O/I*_) (Fig. 4).

Finally, we tested two extreme combinations of CA shape and flow rate in order to estimate the maximum achievable Δ*P*_*net*_ using the collected physiological data. Since we expect the net pressure difference to arise mainly due to flow rate asymmetries, the flow rate of highest asymmetry (within the investigated group) was applied to the subject geometry with the smallest CA area (Config. 1). We also applied the flow of largest SV to this geometry (Config. 2). In separate simulations, the net flow was added to these configurations to estimate its potential contributions, using the group average of 0.77 mL/min.

### Statistics

Statistical calculations were performed using MATLAB (version R2017b, The Mathworks, Natick, MA). The existence of a Δ*P*_*net*_ was tested by a two-sided t-test, as were the bench-model comparisons (significance level α = 0.05). A *positive* pressure difference corresponded to higher pressure in the third ventricle. The relation between Δ*P*_*net*_ and the different flow rate and geometrical characteristics were analyzed using a generalized linear model (GLM). The GLM was carried out with Δ*P*_*net*_ as the dependent variable and CA area, SV and the asymmetry ratio (*Q*_*O/I*_ or *r*_*O/I*_) as random continuous predictor variables. We used only one asymmetry ratio at a time, resulting in two separate GLMs. Values are presented as mean $\pm$ SD unless otherwise specified.

## Results

Based on aqueductal SV the ICC for the PCMRI analysis was *r *= 0.996, with CI = [0.963–0.999] (two-way random effects, absolute agreement, single rater/measurement), indicating excellent agreement.

### The bench-model test

The bench-test results verified a positive Δ*P*_*net bench*_ over the cardiac cycle, an effect that was magnified for the CSF flow from INPH compared to healthy (Δ*P*_*net bench*_: 24.0 ± 1.0 vs. 5.0 ± 1.7 Pa, p < 0.01). The CFD simulations closely recreated the bench-test results (Δ*P*_*net bench CFD*_: 25.1 ± 2.3 vs. 6.4 ± 0.2 Pa, p < 0.01).

### Patient-specific pressure difference

**Fig. 5:**
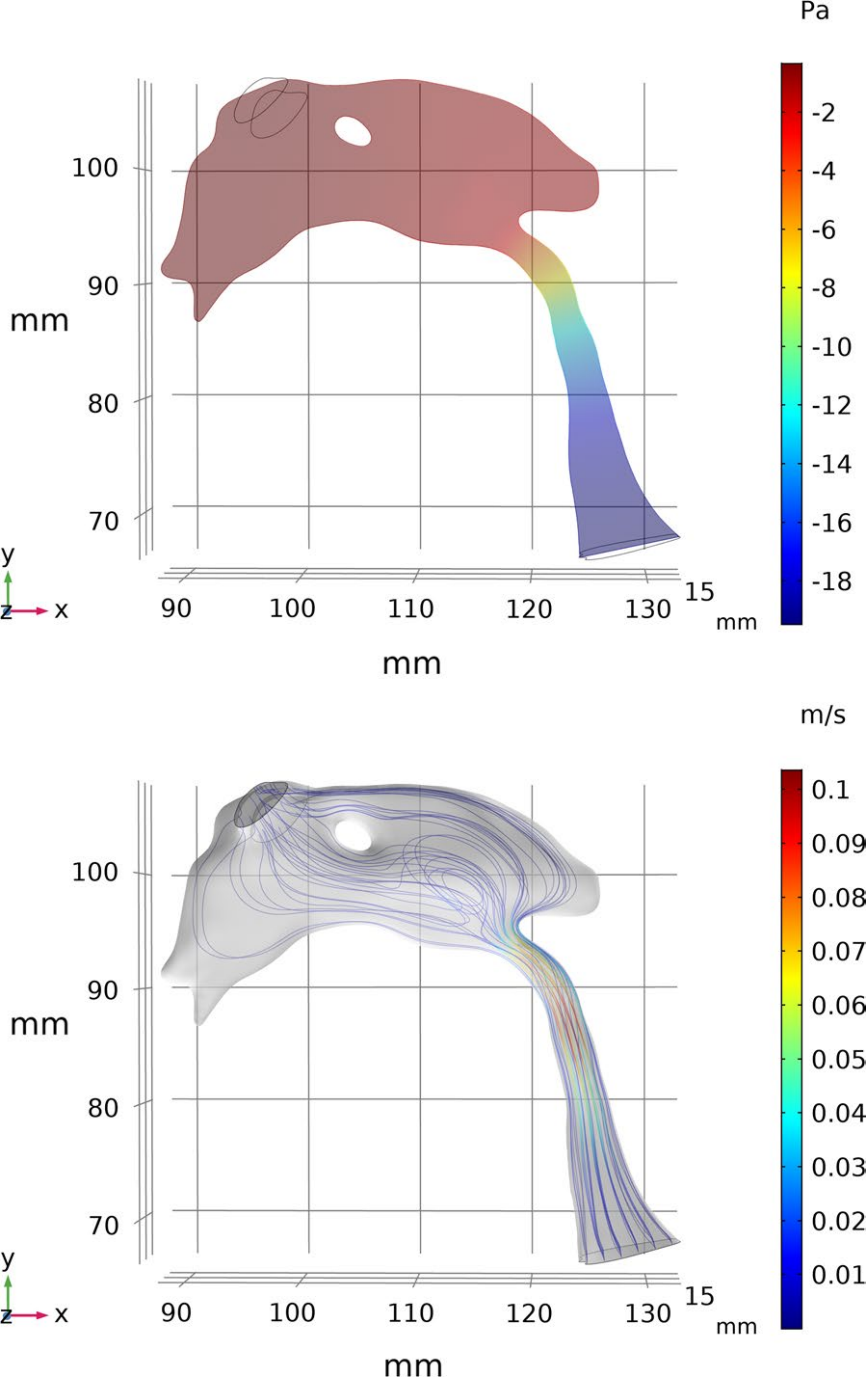
This figure shows the pressure and velocity distributions for a typical subject. Upper: a sagittal slice of the pressure distribution for the point in time of largest pressure difference. The change in pressure occurs almost entirely over the CA, with only small changes in the third and fourth ventricle. Lower: velocity streamlines during maximum outflow. The highest velocities were observed in the CA

**Fig. 6:**
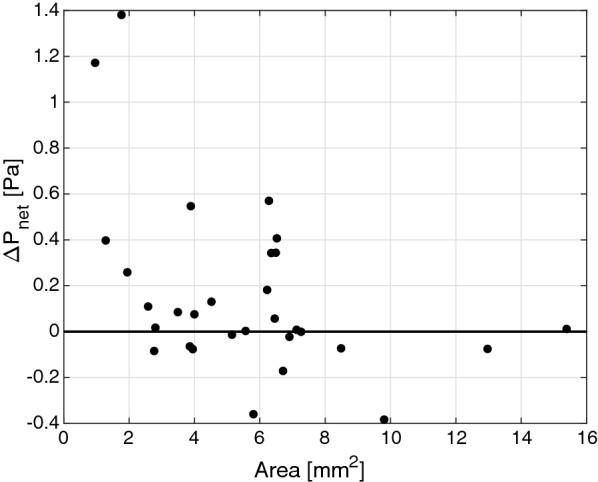
The Δ*P*_*net*_ as a function of CA cross-sectional area. Only some of the very smallest aqueducts achieved a pressure difference close to or above 1 Pa

An example image of the pressure and velocity distributions for a typical simulation is shown in Fig. 5. The Δ*P*_*net*_ due to CSF pulsations was found to be non-zero for the study group (p = 0.03) with a positive magnitude of 0.2 ± 0.4 Pa (0.001 ± 0.003 mmHg), indicating higher pressure in the third ventricle (range 1.4 to − 0.4 Pa). The maximum pressure difference over the cardiac cycle Δ*P*_*max*_ was 20.3 ± 11.8 Pa (0.15 ± 0.09 mmHg) and occurred during systole. We found no correlation between Δ*P*_*net*_ and ventricular size based on Evan’s index (Pearson’s correlation coefficient r = − 0.01, p = 0.95). Individual results for the Δ*P*_*net*_ are shown in Fig. 6 as a function of CA cross-sectional area. Roughly two-thirds of the subjects had a positive Δ*P*_*net*_. Looking only at the INPH subgroup the Δ*P*_*net*_ only approached trend level: Δ*P*_*net*_ = 0.1 ± 0.4 Pa (p = 0.14, n = 15, 10/15 had a positive Δ*P*_*net*_) and Δ*P*_*max*_ = 20.1 ± 8.4 Pa. If only the 6 subjects not primarily diagnosed with hydrocephalus were removed, the statistical significance also vanished Δ*P*_*net*_ = 0.1 ± 0.3 Pa (p = 0.10, n = 24).

The GLM analysis showed that Δ*P*_*net*_ was significantly associated with CA cross-sectional area (p = 0.01) and the maximum flow asymmetry ratio *Q*_*O/I*_ (p = 0.04), while no association was found for aqueductal SV (p = 0.35). Replacing *Q*_*O/I*_ with the time asymmetry ratio *r*_*O/I*_ in the GLM resulted in a non-significant association for *r*_*O/I*_ (p = 0.10), while the results for the CA area and SV were unchanged. *r*_*O/I*_ was, however, strongly correlated with *Q*_*O/I*_ (Pearson’s correlation coefficient r = − 0.88, p < 0.001).

The results for the extreme cases are presented in Table 3; both of the investigated configurations yielded pressure differences of similar magnitude. The maximum Δ*P*_*net*_ achieved was 4.9 Pa, corresponding to the geometry with smallest CA cross-sectional area combined with the largest SV (i.e. Config. 2). Adding the group average net flow to this configuration increased Δ*P*_*net*_ to 9.0 Pa. The diameter of the CA used for the extreme cases was 1.1 mm.

**Table 3:** Resulting Δ*P*_*net*_ (presented in Pa) for two extreme combinations of CA flow and cross-sectional area

	Config. 1	Config. 2
Pulsations only	3.2	4.9
Net flow only	0.8	0.8
Pulsations + net flow	5.5	9.0

Configuration 1 corresponds to the CA with the smallest area subjected to the CA flow with the largest flow asymmetry. Configuration 2 corresponds to the CA with the smallest area subjected to the CA flow of largest SV

The raw MRI data are summarized in Table 4. The aqueductal SV was positively correlated with the cross-sectional area of the aqueduct (Pearson’s correlation coefficient r = 0.37, p = 0.04). Only four subjects had a CA net flow in the cranial direction, three of them were diagnosed as INPH. The Reynolds numbers in the aqueducts were 108 ± 63 at the time of maximum outflow from the ventricles, supporting the assumption of laminar flow.

**Table 4:** Results of the PC-MRI and FIESTA measurements in the CA

Parameter	Mean $\pm$ SD (n = 30)
SV (μL)	80 ± 51
Evan’s index	0.37 ± 0.04
Cross-sectional area (mm^2^)	5.6 ± 3.2
Net flow (mL/min)	0.74 ± 0.71^a^
Max flow (caudal) (mL/min)	21.6 ± 11.8^a^
Min flow (cranial) (mL/min)	− 15.9 ± 9.8^a^
Max flow-min flow (mL/min)	37.5 ± 21.3
Maximum flow ratio (*Q*_*O/I*_)	1.32 ± 0.28
Time ratio *r*_*O/I*_	0.85 ± 0.15
Net flow direction (+/−)^a^	26/4

^a^Positive sign indicates flow out from the ventricles

## Discussion

In this study we investigated the hypothesis that aqueductal flow pulsations could introduce net pressure effects across the open CA that could source ventriculomegaly in communicating hydrocephalus. The investigation consisted of numerically calculating the pressure difference Δ*P* across the CA using CFD based on high resolution MRI data from patients investigated for communicating hydrocephalus. We found that the CSF flow pulsations over the communicating CA did generate net pressure effects over the cardiac cycle, but of very low magnitude.

A relationship between high pulsations and a large ventricular volume has been shown previously [26, 27], but the question has remained whether the pulsations are a cause or merely a result of the ventriculomegaly. Our results indicate that the CSF pulsations over the CA work in favor of a transmantle pressure gradient, but only with an average pressure difference of 0.2 Pa. Our peak-to-peak change in Δ*P* of $\sim$ 30 Pa was associated with a change in ventricular volume of 80 μL (our SV). Using that scaling, a Δ*P* of 0.2 Pa compared to a Δ*P* of 0 Pa would translate to a change in ventricular volume by 0.5 μL (less than 10^−5^ of the total ventricular volume), which is minute. The magnitude of 0.2 Pa is also minimal when compared to the predicted transmantle pressure difference of 235.44 Pa (1.764 mmHg) required to explain more acute cases of (normal pressure) hydrocephalus [16]. Furthermore, forty percent of our subjects had an estimated net pressure difference in the opposite direction (i.e. higher pressure in the fourth ventricle and subarachnoid space), which would work against an enlargement of the third and lateral ventricles in those subjects. The lack of a significant Δ*P*_*net*_ when removing the patients diagnosed with diseases other than hydrocephalus (n = 6) also suggest that the magnitude of Δ*P*_*net*_ may not be relevant in communicating hydrocephalus, as did the results for the INPH subgroup. The largest Δ*P*_*net*_ was observed among the smallest CAs (Fig. 6), which could indicate that a narrowed or semi-obstructed CA is required to generate any substantial magnitude of this pressure difference. For these reasons, we deem it unlikely that the CA pulsations source ventriculomegaly through a pressure difference from flow through the CA. However, since we cannot explicitly determine the long-term effects in this study, we cannot rule out the possibility that the Δ*P*_*net*_ (even of the magnitude observed) could affect the ventricular system over very long periods of time.

We should note that this study did not take disease progression into account, and it is possible that the effects we were looking for had already been compensated for, e.g. by an increase in CA area, and that the pressure difference may have been larger at an earlier stage of the disease. The results from our analysis of the extreme configurations (Table 3) show that with a small CA, a Δ*P*_*net*_ close to 5 Pa could be achieved by either a severe flow asymmetry or a small asymmetry and an increased SV. With the additional contribution from the net flow, the Δ*P*_*net*_ could be as big as $\sim$ 10 Pa. This is also supported by the experimental model results, where a net pressure of 24 Pa was observed across the model CA (the 1 mm model diameter was slightly smaller than the 1.1 mm CA used for the extreme cases). To investigate whether the pressure effects are diminished over time, and how Δ*P*_*net*_ may change during the development of the disease, longitudinal studies would be needed. Such studies would likely demand data from the early stages of the disease and probably before the symptoms appear, which requires population studies.

The results for the GLM verified the association between Δ*P*_*net*_ and CA flow asymmetry, in addition to the cross-sectional area. Thus, the results support the biomechanical principles behind the hypothesized Δ*P*_*net*_. The one exception to this is the lack of association between the SV and Δ*P*_*net*_, something we expected to find, especially due to the strong correlation between CA cross-sectional area and SV observed previously [27]. Since the correlation between CA area and SV was not as strong in our study, and the range of the CA area was much smaller, it could mean that this relationship is altered with disease progression.

It is important to stress that the asymmetrical pressure effects are not only dependent on the asymmetric flow but also on the asymmetry in the shape of the CA inlet and outlets, i.e. where the CA transitions into the third and fourth ventricle respectively (the locations where the discharge effects are expected to be the largest). Thus accurately capturing these effects requires use of the entire CA geometry, hence the focus on optimizing the geometrical data.

We did not include wall movement of the ventricles, which may slightly affect the resulting CA pressure difference [28]. Furthermore, the use of the CA volumetric flow curve as input in the fourth ventricle may introduce small changes to the pressure difference. Also, we did not include respiratory gating in this study. A recent study by Spijkerman et al. [29] has shown that net flow CA measurements can be confounded by respiratory effects, which further indicates that PCMRI CA net flow results must be interpreted with caution. However, respiration does not seem to affect SV [29], which motivates the choice in our study of looking at only the contribution from the pulsations, while removing the effects of net flow. The measurements were only performed in the supine posture, even though patients spend most of their time in an upright position. Thus, the alteration in *ΔP*_*net*_ with posture was not determined in this study. It is also likely that we are pushing the limit for the minimum pressure magnitudes that can be correctly detected using input data to CFD from MRI techniques available today.

To ascertain the reliability of our CFD approach we put a lot of effort into optimizing the input data to the simulations, achieving a PCMRI resolution that is far above the average [30]. In addition, CFD simulations were evaluated against bench tests. Even though the model geometry was highly simplified in these bench tests, the results indicate that our CFD approach can accurately estimate discharge effects for pulsatile flows through aqueduct-like enlargements/contractions. Furthermore, the maximum difference in CA velocity between the CFD and the MRI data (Fig. 6) was generally small, with only a few subjects showing larger deviations (the flow rates were identical by definition). Finally, the pressure differences observed in this study are comparable to that found in previous CFD case studies of hydrocephalus [9, 18], with the maximum difference (i.e. Δ*P*_*max*_) being $\sim$ 20–30 Pa. It is interesting to note that both the net and maximum effects in the current study are slightly larger compared to that calculated in healthy subjects [10] (roughly five times larger), which could indicate that the pulsations in hydrocephalus may have an effect on the transmantle pressure gradient, but one that is still very small in magnitude.

## Conclusions

This study confirmed that a transmantle pressure difference (Δ*P*_*net*_) due to CSF pulsations is introduced over the open CA, with higher pressure in the third ventricle, however the magnitude was low ($\sim$ 0.2 Pa). While this transmantle pressure difference from CA flow is likely too small to explain the ventriculomegaly in communicating hydrocephalus, long-term cohort or modeling studies must be performed to assess its cumulative effects on the ventricles.

## Data Availability

The datasets used and/or analysed during the current study are available from the corresponding author on reasonable request.

## Abbreviations

CA: cerebral aqueduct
CFD: computational fluid dynamics
CSF: cerebrospinal fluid
FIESTA: fast imaging employing steady state acquisition
GLM: generalized linear model
ICC: intraclass correlation coefficient
INPH: idiopathic normal pressure hydrocephalus
PCMRI: phase contrast magnetic resonance imaging
ROI: region of interest
SV: aqueductal stroke volume
Δ*P*_*net*_: net pressure difference across the cerebral aqueduct over the cardiac cycle
Δ*P*_*max*_: maximum pressure difference across the cerebral aqueduct
*Q*_*O/I*_: the maximum ventricular outflow/inflow ratio through the aqueduct
*r*_*O/I*_: the ratio of time duration of ventricular outflow vs. inflow through the aqueduct

## Footnotes

1 PCMRI measurements were also performed at the inlet plane in the fourth ventricle, but these measurements were discarded due to low signal to noise ratios resulting from the large spatial variation in velocities over this plane.

## References

[1] Rekate HL (2009). A contemporary definition and classification of hydrocephalus. Semin Pediatr Neurol.

[2] Mascalchi M, Ciraolo L, Tanfani G, Taverni N, Inzitari D, Siracusa GF (1988). Cardiac-gated phase MR imaging of aqueductal CSF flow. J Comput Assist Tomogr.

[3] Abbey P, Singh P, Khandelwal N, Mukherjee KK (2009). Shunt surgery effects on cerebrospinal fluid flow across the aqueduct of Sylvius in patients with communicating hydrocephalus. J Clin Neurosci.

[4] Balédent O, Gondry-Jouet C, Meyer M-E, De Marco G, Le Gars D, Henry-Feugeas M-C (2004). Relationship between cerebrospinal fluid and blood dynamics in healthy volunteers and patients with communicating hydrocephalus. Invest Radiol.

[5] Greitz D, Hannerz J, Rahn T, Bolander H, Ericsson A (1994). MR imaging of cerebrospinal fluid dynamics in health and disease. On the vascular pathogenesis of communicating hydrocephalus and benign intracranial hypertension. Acta Radiol.

[6] Di Rocco C, Pettorossi VE, Caldarelli M, Mancinelli R, Velardi F (1978). Communicating hydrocephalus induced by mechanically increased amplitude of the intraventricular cerebrospinal fluid pressure: experimental studies. Exp Neurol.

[7] Di Rocco C, Di Trapani G, Pettorossi VE, Caldarelli M (1979). On the pathology of experimental hydrocephalus induced by artificial increase in endoventricular CSF pulse pressure. Childs Brain.

[8] Pettorossi VE, Di Rocco C, Mancinelli R, Caldarelli M, Velardi F (1978). Communicating hydrocephalus induced by mechanically increased amplitude of the intraventricular cerebrospinal fluid pulse pressure: rationale and method. Exp Neurol.

[9] Sweetman B, Xenos M, Zitella L, Linninger AA (2011). Three-dimensional computational prediction of cerebrospinal fluid flow in the human brain. Comput Biol Med.

[10] Bardan G, Plouraboue F, Zagzoule M, Baledent O (2012). Simple patient-based transmantle pressure and shear estimate from cine phase-contrast MRI in cerebral aqueduct. IEEE Trans Biomed Eng.

[11] Qvarlander S, Ambarki K, Wåhlin A, Jacobsson J, Birgander R, Malm J (2017). Cerebrospinal fluid and blood flow patterns in idiopathic normal pressure hydrocephalus. Acta Neurol Scand.

[12] Oliveira PJ, Pinho FT, Schulte A (1998). A general correlation for the local loss coefficient in Newtonian axisymmetric sudden expansions. Int J Heat Fluid Flow.

[13] Bullen PR, Cheeseman DJ, Hussain LA, Ruffellt AE (1987). The determination of pipe contraction pressure loss coefficients for incompressible turbulent flow. Int J Heat Fluid Flow.

[14] Stephensen H, Tisell M, Wikkelsö C, Hodge CJ, Gjerris F, Børgesen SE (2002). There is no transmantle pressure gradient in communicating or noncommunicating hydrocephalus. Neurosurgery.

[15] Eide PK, Sæhle T (2010). Is ventriculomegaly in idiopathic normal pressure hydrocephalus associated with a transmantle gradient in pulsatile intracranial pressure?. Acta Neurochir.

[16] Dutta-Roy T, Wittek A, Miller K (2008). Biomechanical modelling of normal pressure hydrocephalus. J Biomech.

[17] Kurtcuoglu V, Soellinger M, Summers P, Boomsma K, Poulikakos D, Boesiger P (2007). Computational investigation of subject-specific cerebrospinal fluid flow in the third ventricle and aqueduct of Sylvius. J Biomech.

[18] Linninger AA, Xenos M, Zhu DC, Somayaji MR, Kondapalli S, Penn RD (2007). Cerebrospinal fluid flow in the normal and hydrocephalic human brain. IEEE Trans Biomed Eng.

[19] Relkin N, Marmarou A, Klinge P, Bergsneider M, Black PM (2005). Diagnosing idiopathic normal-pressure hydrocephalus. Neurosurgery.

[20] Noble DJ, Scoffings D, Ajithkumar T, Williams MV, Jefferies SJ (2016). Fast imaging employing steady-state acquisition (FIESTA) MRI to investigate cerebrospinal fluid (CSF) within dural reflections of posterior fossa cranial nerves. Br J Radiol.

[21] Hingwala D, Chatterjee S, Kesavadas C, Thomas B, Kapilamoorthy TR (2011). Applications of 3D CISS sequence for problem solving in neuroimaging. Indian J Radiol Imaging.

[22] Wahlin A, Ambarki K, Hauksson J, Birgander R, Malm J, Eklund A (2012). Phase contrast MRI quantification of pulsatile volumes of brain arteries, veins, and cerebrospinal fluids compartments: repeatability and physiological interactions. J Magn Reson Imaging.

[23] Heiberg E, Sjögren J, Ugander M, Carlsson M, Engblom H, Arheden H (2010). Design and validation of Segment—freely available software for cardiovascular image analysis. BMC Med Imaging.

[24] Bloomfield IG, Johnston IH, Bilston LE (1998). Effects of proteins, blood cells and glucose on the viscosity of cerebrospinal fluid. Pediatr Neurosurg.

[25] Behrens A, Lenfeldt N, Qvarlander S, Koskinen LO, Malm J, Eklund A (2013). Are intracranial pressure wave amplitudes measurable through lumbar puncture?. Acta Neurol Scand.

[26] Ringstad G, Emblem KE, Geier O, Alperin N, Eide PK (2015). Aqueductal stroke volume: comparisons with intracranial pressure scores in idiopathic normal pressure hydrocephalus. Am J Neuroradiol.

[27] Chiang WW, Takoudis CG, Lee SH, Weis-McNulty A, Glick R, Alperin N (2009). Relationship between ventricular morphology and aqueductal cerebrospinal fluid flow in healthy and communicating hydrocephalus. Invest Radiol.

[28] Fin L, Grebe R (2003). Three dimensional modeling of the cerebrospinal fluid dynamics and brain interactions in the aqueduct of sylvius. Comput Methods Biomech Biomed Eng.

[29] Spijkerman JM, Geurts LJ, Siero JCW, Hendrikse J, Luijten PR, Zwanenburg JJM (2019). Phase contrast MRI measurements of net cerebrospinal fluid flow through the cerebral aqueduct are confounded by respiration. J Magn Reson Imaging.

[30] Ragunathan S, Pipe JG (2018). Radiofrequency saturation induced bias in aqueductal cerebrospinal fluid flow quantification obtained using two-dimensional cine phase contrast magnetic resonance imaging. Magn Reson Med.

[31] Malm J, Jacobsson J, Birgander R, Eklund A (2011). Reference values for CSF outflow resistance and intracranial pressure in healthy elderly. Neurology.

